# Peripheral blood levels of C-reactive protein in posttraumatic stress disorder: a systematic review and meta-analysis

**DOI:** 10.3389/fpsyt.2026.1839701

**Published:** 2026-07-30

**Authors:** Ling Yang, Jiao Zou, Linghu Cai, Cheng Qian, Hui Jiang, Chaojun Li, Junwei Gao, Xiangyu Chen, Minghua Liu

**Affiliations:** 1 Department of Emergency, Southwest Hospital, Third Military Medical University (Army Medical University), Chongqing, China; 2 Department of Military Cognitive Psychology, School of Psychology, Third Military Medical University (Army Medical University), Chongqing, China; 3 School of Psychology, Third Military Medical University (Army Medical University), Chongqing, China

**Keywords:** C-reactive protein, meta-analysis, peripheral blood, posttraumatic stress disorder, systematic review

## Abstract

**Background:**

Posttraumatic stress disorder (PTSD) was reported to be associated with inflammation. C-reactive protein (CRP) is an important biomarker of systemic inflammation. A series of studies have reported the change of peripheral blood CRP in PTSD. However, the results were controversial. Our aim was to evaluate whether abnormal peripheral blood CRP levels was associated with PTSD using a systematic review and meta-analysis method.

**Methods:**

Five databases (PubMed, Embase, Web of Science, Cochrane library and PsycINFO) were searched for available articles published up to 2 May, 2026. Hedges’ g with its corresponding 95% confidence interval (CI) was selected to assess the group difference in CRP concentrations, a random effects model or fixed effects model were selected according to the results of heterogeneity test.

**Results:**

A total of 32 studies (39 reports) with 2667 PTSD patients and 8166 controls were included in our analysis. Significantly elevated peripheral blood CRP levels were found in PTSD participants compared with controls (Hedges’ g = 0.188, 95% CI 0.080 to 0.296, p = 0.001). Subgroup analysis showed a significant elevation in serum (Hedges’ g = 0.208, 95% CI 0.049 to 0.368, *p* = 0.01) and the female groups (Hedges’ g = 0.446, 95% CI 0.043 to 0.849, *p* = 0.03), but not in plasma (Hedges’ g = 0.140, 95% CI –0.051 to 0.331, *p* = 0.151) and the male groups (Hedges’ g = 0.143, 95% CI –0.008 to 0.294, *p* = 0.063), suggesting that the type of biological sample and gender may influence the observed association. Other subgroup analyses (e.g., control type, PTSD type, detection method and traumatic type) generally showed consistent results. The significance of the association between peripheral blood CRP concentration and PTSD remained unchanged after excluding studies outside the Galbraith plot. Baujat plot identified three studies contribute most to the heterogeneity. Removing any single study verified by sensitivity analysis did not reverse the association. Meta-regression analysis revealed that the association was moderated by mean body mass index (BMI). No obvious publication bias was found in the meta-analysis.

**Conclusion:**

This meta-analysis provides evidence for elevation of peripheral blood CRP in PTSD. However, due to the lack of data on time since trauma in the included studies, the current findings cannot differentiate whether the elevated CRP is an acute phase response to the traumatic event itself or the inflammatory state of chronic PTSD. More well-designed longitudinal studies are required to demonstrate this conclusion in the future.

## 1 Introduction

Posttraumatic stress disorder (PTSD) is a severe psychiatric disorder caused by intense psychological stress resulting from sudden catastrophic events or natural disasters. It is commonly observed among high-risk populations like survivors of concentration camp, victims of natural disasters, and military veterans. This disorder can trigger symptoms including traumatic re-experiencing, heightened alertness, avoidance or numbness in patients (1, 2). In addition to these typical abnormal psychological behaviors, PTSD patients usually experience comorbid immune-related conditions, such as cardiovascular and autoimmune disorders (3, 4).

Growing evidences suggested that inflammation may serve as a potential mechanism underlying the development of PTSD (5). Researches have demonstrated that individuals with PTSD often exhibit elevated levels of various inflammatory markers, including C-reactive protein (CRP), interleukin-6 (IL-6), and tumor necrosis factor-alpha (TNF-α) (6, 7). CRP, synthesized by hepatocytes in response to pro-inflammatory cytokines, is the most prevalent early inflammatory biomarker. CRP serves as a crucial indicator for assessing both the type of infection and the severity of inflammation. In cases of acute inflammation, severe bacterial infection, or tissue damage, CRP levels will be detectable within 6–8 hours and peak within 24–48 hours (8). By compromising blood-brain barrier (BBB) integrity and initiating secondary inflammatory signaling within the cerebral endothelium, peripheral CRP ultimately triggers glial cell activation and impairs central nervous system (CNS) function (9). Evidence showed that there was a correlation between PTSD diagnosis and elevated levels of peripheral blood CRP (10–12). A study conducted on community residents found that patients with PTSD exhibit nearly twofold higher levels of serum CRP compared to those without PTSD (11). In comparison with healthy controls, female PTSD patients exhibited significantly elevated plasma levels of CRP and IL-6; however, following recovery, these patients’ CRP and IL-6 levels decreased and approached those of the non-trauma control group (13). The single nucleotide polymorphism (SNP) of the CRP gene was also closely associated with both serum CRP and the severity of PTSD, and there was a positive correlation between CRP levels and PTSD symptoms (14). According to a cross-sectional study, the concentrations of serum CRP were significantly elevated in veterans with PTSD compared to controls (15). Another study revealed higher serum CRP levels in the PTSD group than those in the non-PTSD group; nevertheless, this association no longer significant after adjusting for body mass index (BMI) and triglycerides; furthermore, no significant correlation was observed between SNPs of serum CRP and either PTSD or CRP levels (16). Consequently, despite numerous clinical studies investigated the association between peripheral blood CRP levels and PTSD, the results were inconsistent.

Therefore, in the current review we systematically assessed the accumulative effects between peripheral blood CRP and PTSD patients in a larger sample size set by meta-analysis. We also accounted for factors including categorical variable (i.e., control sample type, PTSD sample type, gender type, blood fraction, detection method, and traumatic type), continuous variables (i.e., sample size, % female, mean age, mean BMI, smoking status, alcohol use, antidepressant use, comorbid depression, and comorbid somatic diseases) that may influence the results. We hypothesized that there was an association between elevated peripheral blood CRP and PTSD.

## 2 Materials and methods

The systematic review and meta-analysis followed the guidance in the Preferred Reporting Items for Systematic Reviews and Meta-Analyses (PRISMA) statement (17). The study had also been prospectively registered in PROSPERO, with the registration number CRD42024554449.

### 2.1 Search strategy

A literature search was conducted in the following databases: PubMed, Embase, Web of Science, Cochrane library and PsycINFO. The initial search covered the period from inception to 26 April 2024. To incorporate the most recent evidence, the search was updated to include literatures published up to 2 May 2026. The search strategy utilized the following terms: ([“post-traumatic stress disorder”] OR [“traumatic stress”] OR [“psychological trauma”] OR [“PTSD”]) AND ([“C-Reactive Proteins”] OR [“CRP”]). Furthermore, reference lists of all included papers were manually retrieved for potential eligibility studies. All selected literatures were imported into Endnote X9 software for further analysis. Two authors (LY and ZJ) independently searched the literatures and selected the eligible studies.

### 2.2 Eligibility criteria

Studies were considered eligible if they met the following criteria: (1) original studies that diagnosed PTSD using well-validated diagnostic criteria; (2) studies compared peripheral blood CRP levels between patients with PTSD and healthy controls; (3) studies reported CRP levels as mean ± standard deviation (SD) or provided sufficient data that can be converted to mean ± SD. The following exclusion criteria were applied: (1) studies with irrelevant or insufficient data; (2) *in vitro* or animal studies; (3) reviews, conference abstracts, meta-analysis, comments, case reports, or letters; (4) studies with CRP data after pharmacological stimulation. When longitudinal studies were included, only CRP data from the latest time point were extracted to reflect the most recent inflammatory status. In case where articles had overlapping data, only the study with the largest sample size was included in the analysis.

### 2.3 Data extraction and quality assessment

The following data were independently extracted from each study by two reviewers (LY and LC) using a standardized spreadsheet: the first author, publication year, country, diagnostic criteria for PTSD, type of blood fraction, detection method, type of control, the type of traumatic events, sample size, gender ration, age, and BMI, and, if reported, smoking status, alcohol use, antidepressant use, comorbid depression, and comorbid somatic diseases (e.g., hypertension, diabetes, cardiovascular disease). For studies reporting mean and standard errors (SE) or median with interquartile ranges, the data were transformed using the formula SE = SD/√N (N = sample size) or Shi et al’s method (18). When data were only available in diagrammatic format, they were extracted using Origin software (2018). Disagreements were resolved by discussion or arbitration with GJ, XC or ML.

The methodological quality of the included studies was independently determined using the Newcastle–Ottawa scale (NOS), which consist of three groups: the selection of study groups, the comparability of groups, and the outcome measure (19).

### 2.4 Statistical analysis

Comprehensive meta-analysis version 3.0 (CMA3.0) software, Stata 17.0 software and a ‘meta’ package in R (version 4.5.1) were employed for data synthesis. The Hedges’ g and its corresponding 95% confidence interval (CI) were used to evaluate the cumulative effect size of the association between CRP concentration and PTSD (20). Cochran’s Q statistics and *I*^2^ were utilized to assess heterogeneity, when Q statistic (*p* < 0.1) or *I*^2^ > 50%, a random effects model was applied, otherwise, a fixed effects model was used (21). The possible source of heterogeneity was identified by performing subgroup analyses according to the control sample type, PTSD sample type, gender, blood fraction, detection methods, and the traumatic type. Galbraith plot was conducted to detect the heterogeneity and remove outlier studies (22). Baujat plot was additionally constructed to visually identify individual studies that contributed most to the overall heterogeneity and the overall effect (23). Meta-regression analyses were used to explore whether continuous variable (e.g., sample size, % female, mean age, mean BMI) moderated the results (24). Sensitivity analysis was performed using the leave-one-out method to test the stability of the primary results. Publication bias was assessed through visual inspection of funnel plot when the number of eligible studies was ten or more (25). Egger’s test further examined asymmetry of the funnel plot, and *p* < 0.05 was considered statistically significant, then a trim-and-fill method would be used to adjust the overall effect estimate (26).

## 3 Results

### 3.1 Literature search

**Figure 1:**
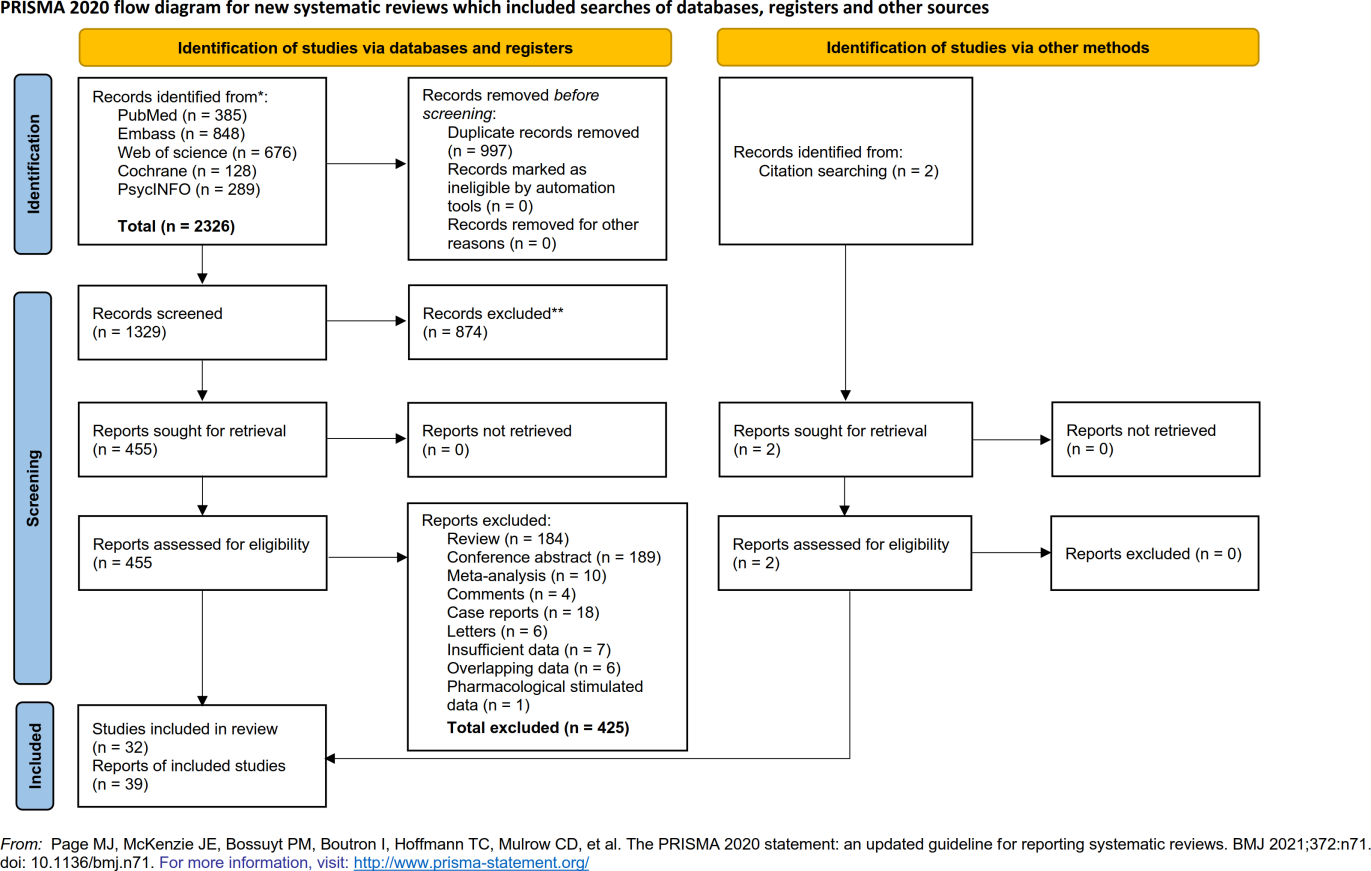
Flow chart of the study selection process. *Consider, if feasible to do so, reporting the number of records identified from each database or register searched (rather than the total number across all databases/registers). **If automation tools were used, indicate how many records were excluded by a human and how many were excluded by automation tools.

An initial literature search identified 2326 publications from five databases, of which 385 were from PubMed, 848 were from Embase, 676 were from Web of Science, 128 were from Cochrane library, and 289 were from PsycINFO. After removing 997 duplications and excluding 874 irrelevant studies, a total of 455 records were identified as potential eligible studies. Following full-text reading, we further excluded 425 articles for various reasons (184 reviews, 189 conference abstracts, 10 meta-analyses, 4 comments, 18 case reports, 6 letters, 7 studies with insufficient data, 6 studies with overlapping data, and one study with pharmacological stimulated data). Ultimately, 30 original studies were retained (11, 13, 15, 16, 27–52). Additionally, two studies were identified through references reading (53, 54). In the end, 32 studies (39 reports) were included in the meta-analysis (**Figure 1**).

### 3.2 Study characteristics

As shown in **Table 1**, all the retained studies were conducted between 2004 and 2025, involving 2667 individuals with PTSD and 8166 controls. Among them, six studies were published within the past five years. Of the retained studies, 17 were conducted in the US, 2 each in Germany, the UK, Switzerland, and one each in Japan, Australia, Korea, Nepal, Sweden, Iraq, Croatia, Russia and Bosnia and Herzegovina. Additionally, eight studies assessed CRP levels among female participants and eleven studied male patients. Furthermore, 22 studies reported CRP levels from serum sample, 11 reported from plasma sample. Moreover, 19 studies used nephelometry to determined CRP levels. About the selection of control groups, 12 studies chose non-trauma-exposed control (NTC), 27 studies selected trauma-exposed controls (TC). The included studies covered various ethnicities including the Caucasian race, Asians and Africans. The methodological scores ranged from 5 to 8.

**Table 1:** Main characteristics of included studies.

Reference	PTSD diagnosis	Blood fraction	Detection method	Type of controls	Type of trauma	Sample size		Gender, Female/male		Age, Mean ± SD, years		BMI, Mean ± SD, kg/m^2^		NOS Score
						Controls	PTSD	Controls	PTSD	Controls	PTSD	Controls	PTSD	
Otsuka, 2021, Japan	PDS, MINI	Serum	Nephelometry	NTC	Interpersonal violence (majority)	73	57	73/0	57/0	35.1 ± 13.8	39.7 ± 9.3	20.7 ± 2.6	21.5 ± 3.3	7
Maguire, 2021, UK	CAPS-5, PCL-5	Serum	Cytokine arrays	NTC	—	20	20	10/10	11/9	39.0 ± 2.6	41.5 ± 11.0	29.7 ± 7.9	27.9 ± 6.3	6
Mehta, 2020, US	PSS	Plasma	Ultra WR CRP kit	TC	—	38	18	38/0	18/0	40.1 ± 11.5	32.2 ± 5.8	38.8 ± 12.7	32.1 ± 6.9	6
Eswarappa, 2019, US	PCL	—	Nephelometry	TC	War combat	396	170	12/384	15/155	59.3 ± 11.7	57.0 ± 9.39	28.5 ± 5.12	29.9 ± 6.41	7
Miller, 2018, US	CAPS	Serum	Nephelometry	TC	War combat	123	163	12/111	21/142	32.370 ± 8.551	31.860 ± 8.229	—	—	6
Na, 2017, Korea	IES-R-K	Plasma	Nephelometry	TC	—	368	139	0/368	0/139	37.67 ± 8.32	41.39 ± 7.28	24.03 ± 2.17	24.54 ± 2.20	6
Lindqvist, 2014, US	CAPS	Serum	Nephelometry	TC	War combat	51	51	0/51	0/51	33.7 ± 9.0	34.1 ± 8.7	28.3 ± 4.2	29.9 ± 5.1	6
Jergovic, 2015, Croatia	ICD-10	Serum	Latex immunoassay	TC	War combat	32	69	0/32	0/69	45.56 ± 7.24	47.12 ± 5.92	—	—	5
Fajkić, 2017, Bosnia and Herzegovina (study 1)	ICD-10	Serum	Nephelometry	NTC	—	30	30	0/30	0/30	49.66±6.32	48.60±6.32	—	—	5
Fajkić, 2017, Bosnia and Herzegovina (study 2)	ICD-10	Serum	Nephelometry	TC	War combat	30	30	0/30	0/30	49.7±4.72	48.60±6.32	—	—	5
Muhtz, 2011, Germany	PDS	Serum	Standard laboratory methods	TC	War combat	25	25	16/9	16/9	71 ± 2.0	71 ± 2.5	26.8 ± 5.0	25.2 ± 3.5	6
McCanlies, 2011, US	IES-R-K	Plasma	Nephelometry	TC	—	79	32	31/48	12/20	39 ± 7.4	41.4 ± 7.8	27.8 ±4.0	28.6 ± 5.3	6
Spitzer, 2010, Germany	SCID, MMSE, CID-S	Serum	Nephelometry	NTC	—	2994	55	1545/1449	39/16	53.5 ± 15.0	55.0 ± 16.4	27.8 ± 4.8	28.5 ± 5.3	8
Zhang, 2022, US	PCL-17	Serum	Wide-range CRP assay	TC	War combat	415	316	206/209	167/149	42.9 ± 12.6	42.6 ± 9.7	28.35 ± 6.3	28.79 ± 6.1	7
von Känel,2007,Switzerland	CAPS	Plasma	Nephelometry	TC	Accident	14	14	5/9	5/9	33 ± 11	33 ± 10	25.1 ± 4.6	24.8 ± 3.5	5
Sumner, 2017, US (study 1)	DSM-IV PTSD	Plasma	Nephelometry	NTC	—	175	174	175/0	174/0	42.9 ± 4.8	44.0 ± 4.6	25.1 ± 5.5	26.6 ± 6.2	7
Sumner, 2017, US (study 2)	DSM-IV PTSD	Plasma	Nephelometry	TC	—	175	174	175/0	174/0	44.5±4.8	44.0 ± 4.6	25.4 ± 4.9	26.6 ± 6.2	7
Mc,D.Young, 2021, Australia	CAPS-5	Serum	Latex immunoassay	TC	War combat	140	159	0/140	0/159	69.14 ± 0.33	68.37 ± 0.33	28.95 ± 0.36	30.46 ± 0.37	6
Miller, 2017, US	MINI, CAPS-5	Serum	—	TC	War combat	16	16	0/16	0/16	67.8 ± 1.6	67.0 ± 2.8	29.7 ± 5.6	31.5 ± 5.8	6
D'Elia, 2022, UK	CAPS-5	Serum	ELISA	NTC	Sexual violence	41	58	41/0	58/0	28±7.1	24.6±6.8	24.7±3.7	24.7±5.1	5
Neupane, 2017, Nepal (study1)	CIDI	Serum	—	NTC	Traffic accident (majority)	47	32	—	4/28	—	33.1 ± 7.9	—	—	6
Neupane, 2017, Nepal (study2)	CIDI	Serum	—	TC	—	107	32	—	4/28	—	33.1 ± 7.9	—	—	6
Hasan, 2024, Iraq (study1)	PCL-5	Serum	Nephelometry	NTC	—	55	55	55/0	55/0	—	—	—	—	5
Hasan, 2024, Iraq (study 2)	PCL-5	Serum	Nephelometry	TC	Genocide-related events	55	55	55/0	55/0	—	—	—	—	5
Donovan,2017, US	CAPS	—	Nephelometry	TC	—	363	257	8/355	28/229	59.5±12.2	58.0±10.1	28.4±5.3	30.1±6.0	6
Bersani, 2016, US	CAPS	Plasma	Nephelometry	TC	War combat	65	56	0/65	0/56	32.81 ± 8.40	33.91 ± 8.43	28.07 ± 4.65	30.12 ± 5.26	6
Farr, 2015, US	UCLA PTSD scale	Serum	Roche Cobas c311	TC	—	81	40	39/42	27/13	46.0 ± 3.7	44.9 ± 3.5	28.5 ± 1.3	31.3 ± 1.2	7
Turner, 2013, US	CAPS	—	Nephelometry	TC	War combat	433	230	14/419	24/206	58.0 ± 11.6	58.2 ± 10.3	—	—	7
Gill, 2013, US	SCID-IV, PCL	Plasma	Radioimmunoassay	NTC	Physical, sexual assault	24	26	24/0	26/0	34.79 ± 8.50	34.83 ± 8.24	25.88 ± 5.92	25.51 ± 6.92	6
Park, 2017, US	CAPS-IV, PCL-M	—	Nephelometry	TC	War combat	14	14	1/13	2/12	32.2 ± 1.5	34.4 ± 1.7	28.4 ± 1.2	30.1 ± 1.6	5
H, W,2013, US (study1)	CAPS, PCL	Plasma	Chemiluminescent assay system	NTC	War combat	15	15	0/15	0/15	—	—	—	—	6
H, W,2013, US (study2)	CAPS, PCL	Plasma	Chemiluminescent assay system	TC	War combat	15	15	0/15	0/15	—	—	—	—	6
Söndergaard, 2004 Sweden	CAPS	Serum	Nephelometry	TC	War combat	25	38	—	—	18-48^a^	18-48^a^	—	—	5
von Känel, 2010, Switzerland	CAPS	Plasma	Flow cytometric cytokine assay	TC	Myocardial infarction	29	15	5/24	4/11	58.68±6.9	58.38±7.4	28.48±4.3	27.08±2.9	5
Zinchuk,2025, Russia	MMSE	Serum	Beckman Coulter AU 680	TC	—	95	95	79/16	79/16	37.0 ± 14.6	37.0 ± 14.6	—	—	6
Cullins, 2025, US (study 1)	SCID- IV, DSM- 5	serum	—	NTC	—	502	180	271/231	81/99	36.9 ± 13.9	44.5± 11.3	—	—	7
Cullins, 2025, US, (study 2)	SCID- IV, DSM- 5	serum	—	TC	—	610	180	185/425	81/99	42.5 ± 13.1	44.5± 11.3	—	—	7
Xu, 2023, US, (study 1)	CAPS-5	—	CLIA-certified lab	NTC	War combat	235	44	0/235	0/44	31.78 ±6.19	32.51 ±5.78	27.60 ±4.63	27.74 ±4.92	6
Xu,2023, US, (study 2)	CAPS-5	—	CLIA-certified lab	TC	War combat	158	52	0/158	0/52	33.52 ±7.86	33.40 ±7.84	28.35 ±4.28	28.60 ±3.75	6

^a^ Data represented as range; — = not report; SD, standard deviation; PTSD, posttraumatic stress disorder; BMI, body mass index; NOS score, Newcastle-Ottawa Scale score; NTC, non-trauma-exposed controls; TC, trauma-exposed controls; PDS, Posttraumatic Stress Diagnostic Scale; MINI, Mini-International Neuropsychiatric Interview; CAPS, Clinician-Administered PTSD Scale; PCL, PTSD Checklist-Civilian Version; DSM, Diagnostic and Statistical Manual of Mental Disorders; IES, Impact of Events Scale; ICD, International Classification of Diseases; SCID, Structured Clinical Interview for Axis I disorders; CIDI, Composite International Diagnostic Interview; MMSE, Mini Mental State Examination; UCLA, University of California, Los Angeles; ELISA, Enzyme-linked immunosorbent assay. None of the included studies reported the time since trauma to blood collection.

**Supplementary Table 1** summarized minor characteristics that may influence the results across the included studies, including smoking, alcohol use, antidepressant use, comorbid depression, and comorbid somatic diseases. Reporting of these factors varied considerably across studies: approximately 26 studies reported smoking status, 24 reported alcohol use, 14 reported antidepressant use, 27 reported comorbid depression (either as diagnosis or scale scores), and 14 reported comorbid somatic diseases; and very few studies controlled for these key potential confounders. Additionally, the measurement criteria for different indicators (e.g., for alcohol use assessed as drinks/week, alcohol (g/d) or AUDIT score) and the data presentation formats (e.g., n (%), mean ± SD, mean ± SEM or medians (inter - quartile range)) also varied.

### 3.3 Primary analysis

**Figure 2:**
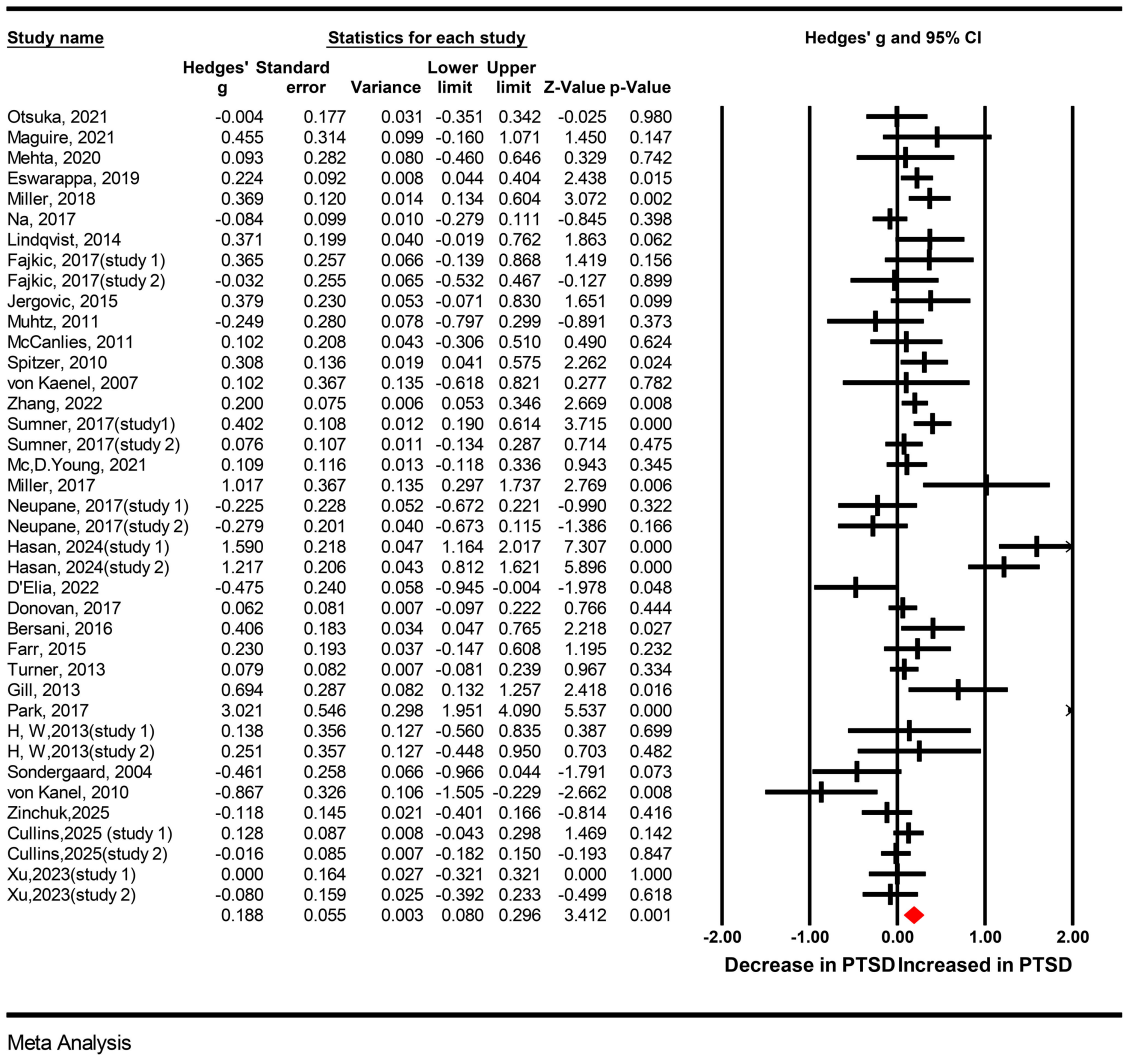
Primary meta-analysis of peripheral blood CRP level in PTSD.

We pooled the data from the 39 reports to evaluate the correlation between peripheral blood CRP levels and PTSD. Due to obvious heterogeneity across studies existed (*I*^2^ = 78.382, *p* < 0.001), a random effects model was selected. The pooled effective size and corresponding 95% CI revealed that levels of peripheral blood CRP were significantly higher in PTSD participants compared to controls (Hedges’ g = 0.188, 95% CI 0.080 to 0.296, *p* = 0.001) (**Figure 2**).

### 3.4 Subgroup analysis

The subgroup analyses of peripheral blood CRP levels of PTSD patients and controls were presented in **Table 2**. Based on the types of controls, peripheral blood CRP concentrations were significantly elevated in individuals with PTSD compared to both NTC (Hedges’ g = 0.274, 95% CI 0.030 to 0.518, *p* = 0.028) and TC (Hedges’ g = 0.149, 95% CI 0.030 to 0.267, *p* = 0.014). Furthermore, veterans with PTSD also exhibited statistically higher CRP concentrations when compared to the control group (Hedges’ g = 0.247, 95% CI 0.103 to 0.391, *p* < 0.001). Moreover, peripheral blood CRP concentrations were markedly increased among female PTSD patients compared to female controls (Hedges’ g = 0.446, 95% CI 0.043 to 0.849, *p* = 0.030), but the difference was not significant among male PTSD patients compared to male controls (Hedges’ g = 0.143, 95% CI −0.008 to 0.294, *p* = 0.063). With respect to blood fraction, a significantly higher effective value was found in serum (Hedges’ g = 0.208, 95% CI 0.049 to 0.368, *p* = 0.010), but not in plasma (Hedges’ g = 0.140, 95% CI −0.051 to 0.331, *p* = 0.151). Additionally, peripheral blood CRP concentration was statistically significantly augmented in PTSD patients compared to controls when using the nephelometry detection method (Hedges’ g = 0.330, 95% CI 0.156 to 0.505, *p* < 0.001). On the other hand, PTSD patients from war combat exhibited obviously elevated peripheral blood CRP levels than corresponding controls (Hedges’ g = 0.203, 95% CI 0.054 to 0.353, *p* = 0.008).

**Table 2:** Summary of subgroup analysis of CRP between PTSD patients and controls. .

Subgroups	Reports	PTSD	Control	Model	Tests of association			Tests of heterogeneity		
	(n)	(n)	(n)		Hedges’ g [95 % CI]	Z-Value	*p*-value	Q value	*p*-value	*I*^2^ (%)
All	39	2667	8166	RE	0.188 [0.080, 0.296]	3.412	0.001	175.778	< 0.001	78.382
Control sample type										
NTC	12	718	4208	RE	0.274 [0.030, 0.518]	2.202	0.028	63.049	< 0.001	82.553
TC	27	2435	3958	RE	0.149 [0.030, 0.267]	2.456	0.014	107.270	< 0.001	75.762
PTSD sample type										
Veteran	16	1319	2111	RE	0.247 [0.103, 0.391]	3.354	0.001	47.466	< 0.001	68.282
Gender type										
Female	8	360	631	RE	0.446 [0.043, 0.849]	2.171	0.030	74.940	< 0.001	90.659
Male	11	561	1123	FE	0.143 [-0.008, 0.294]	1.861	0.063	17.457	0.065	42.718
Blood fraction										
Serum	22	1411	5479	RE	0.208 [0.049, 0.368]	2.561	0.010	115.286	< 0.001	81.784
Plasma	11	489	993	RE	0.140 [-0.051, 0.331]	1.436	0.151	26.848	0.003	62.753
Detection Method										
Nephelometry	19	1520	5527	RE	0.330 [0.156, 0.505]	3.722	< 0.001	123.861	< 0.001	85.468
Traumatic type										
War	16	1428	2181	RE	0.203 [0.054, 0.353]	2.663	0.008	53.308	< 0.001	71.861
Removing studies outside Galbraith plot	29	2372	7271	FE	0.130 [0.072, 0.188]	4.410	< 0.001	33.590	0.215	16.641

RE, random effects model; FE, fixed effects model; NTC, non-trauma-exposed controls; TC, trauma-exposed controls.

### 3.5 Galbraith plot

**Figure 3:**
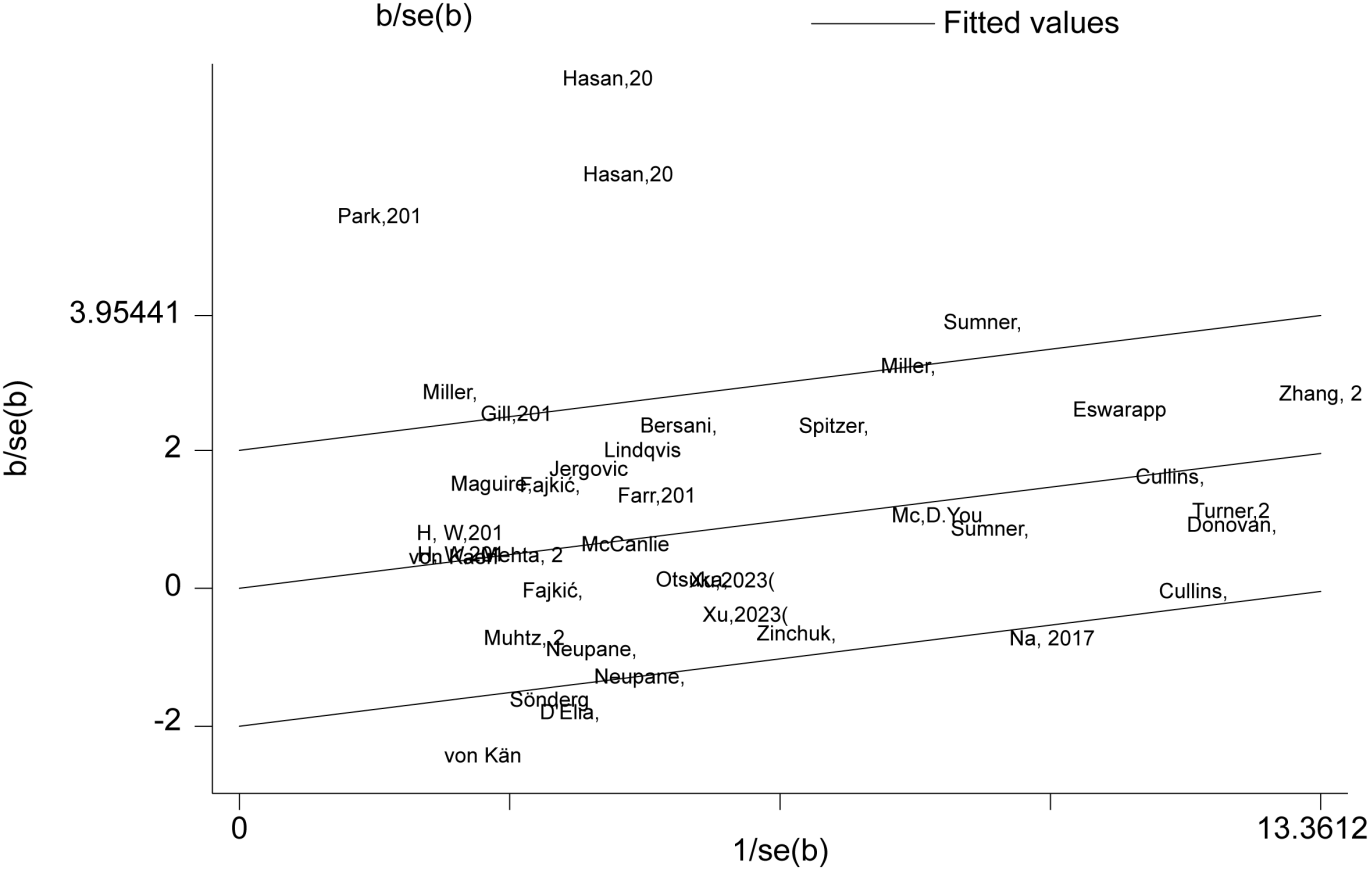
Galbraith plot of peripheral blood CRP level in PTSD patients versus controls.

**Figure 4:**
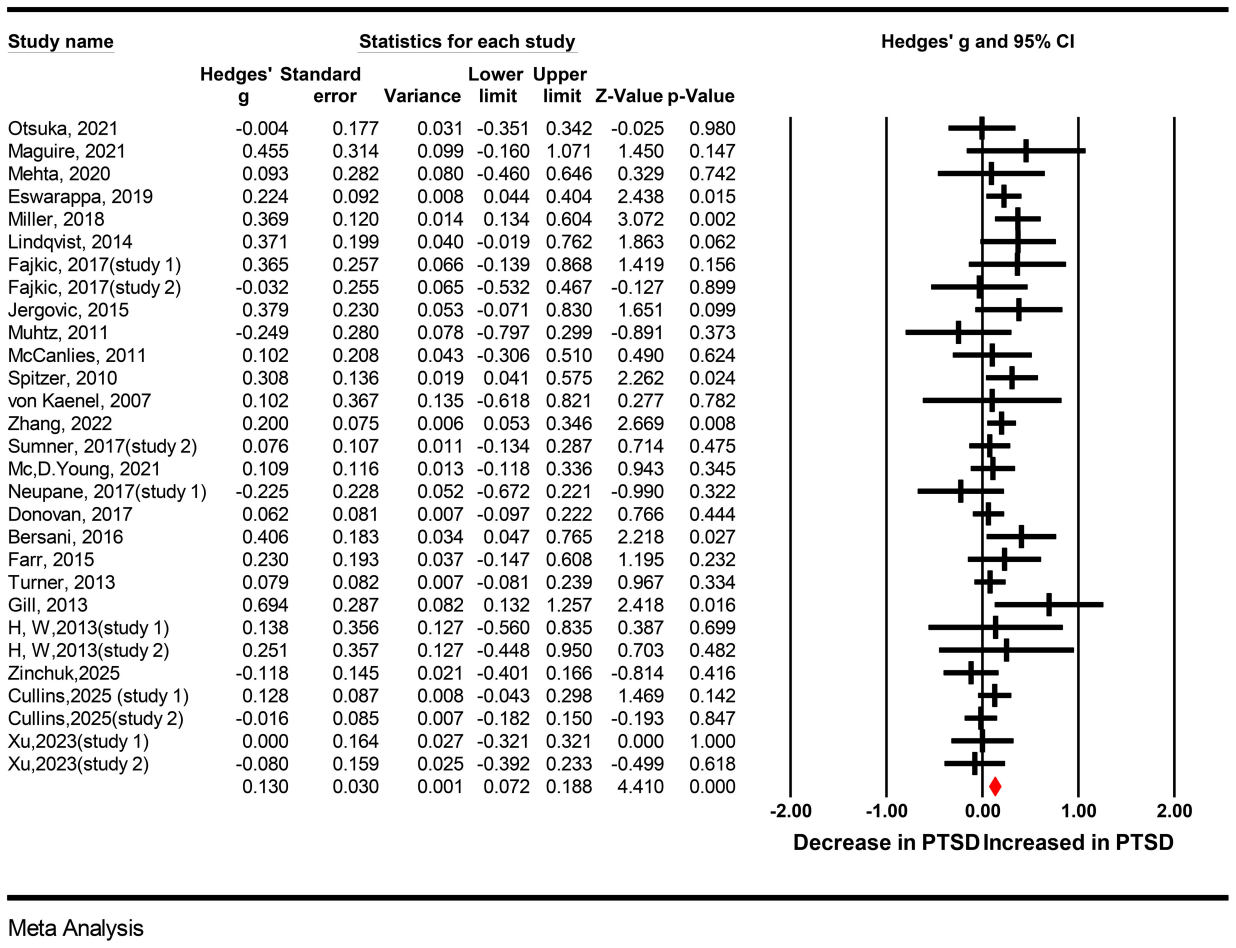
Meta-analysis after removing studies outside Galbraith plot.

To find studies that bring heterogeneity, a Galbraith plot was performed. Ten studies were outside the boundary of Galbraith plot (**Figure 3**) (31, 38–42, 47–49). After excluding these outlier studies, the heterogeneity decreased to 16.641%. The combined effect size remained statistically significant (Hedges’ g = 0.130, 95% CI 0.072 to 0.188, *p* < 0.001), indicating that the results had good stability (**Table 2**; **Figure 4**).

### 3.6 Baujat plot

**Figure 5:**
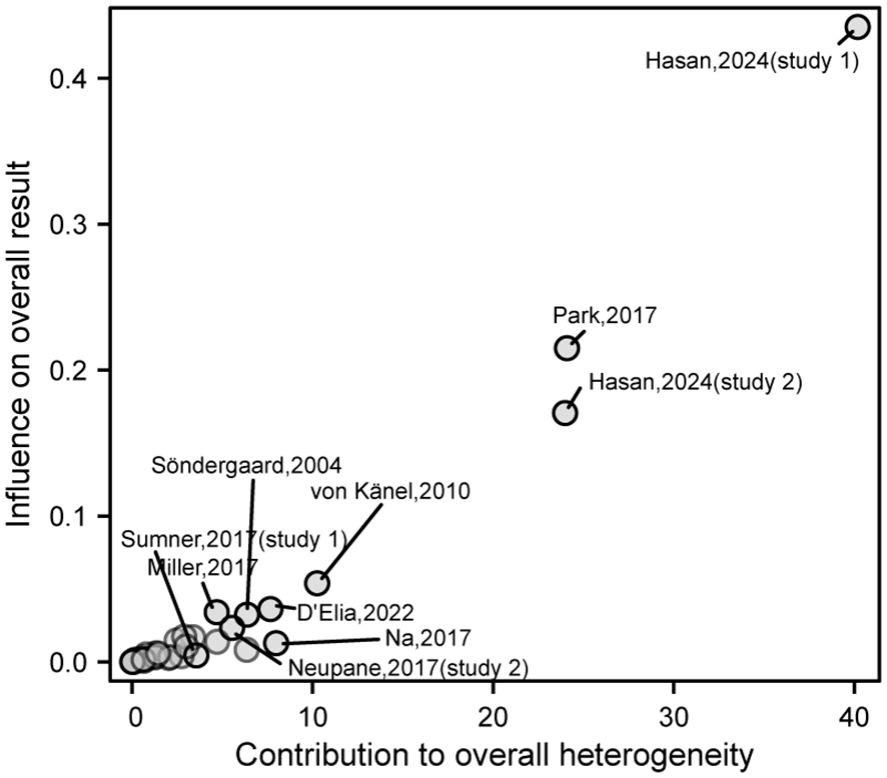
Baujat plot analysis of the included studies on heterogeneity.

Furthermore, the Baujat plot revealed that Hasan, 2024 (study 1), Hasan, 2024 (study 2), and Park, 2017 were all located in the upper-right quadrant of the graph, indicating that these three studies were the most significant contributors to both heterogeneity and the pooled effect size (**Figure 5**) (41, 47). Notably, the studies identified by the Baujat plot as major to heterogeneity overlapped considerably with the 10 studies falling outside the 95% CI boundaries on the Galbraith plot, suggesting the outlier status of these 10 studies.

We further conducted a comprehensive, multi-dimensional comparison of characteristics between these 10 outlier studies and the remaining 24 studies. The results revealed that these outliers exhibited high heterogeneity across various dimensions, with no consistent methodological flaws or systematic design differences (**Supplementary Tables 2**, **3**).

### 3.7 Regression analysis

**Figure 6:**
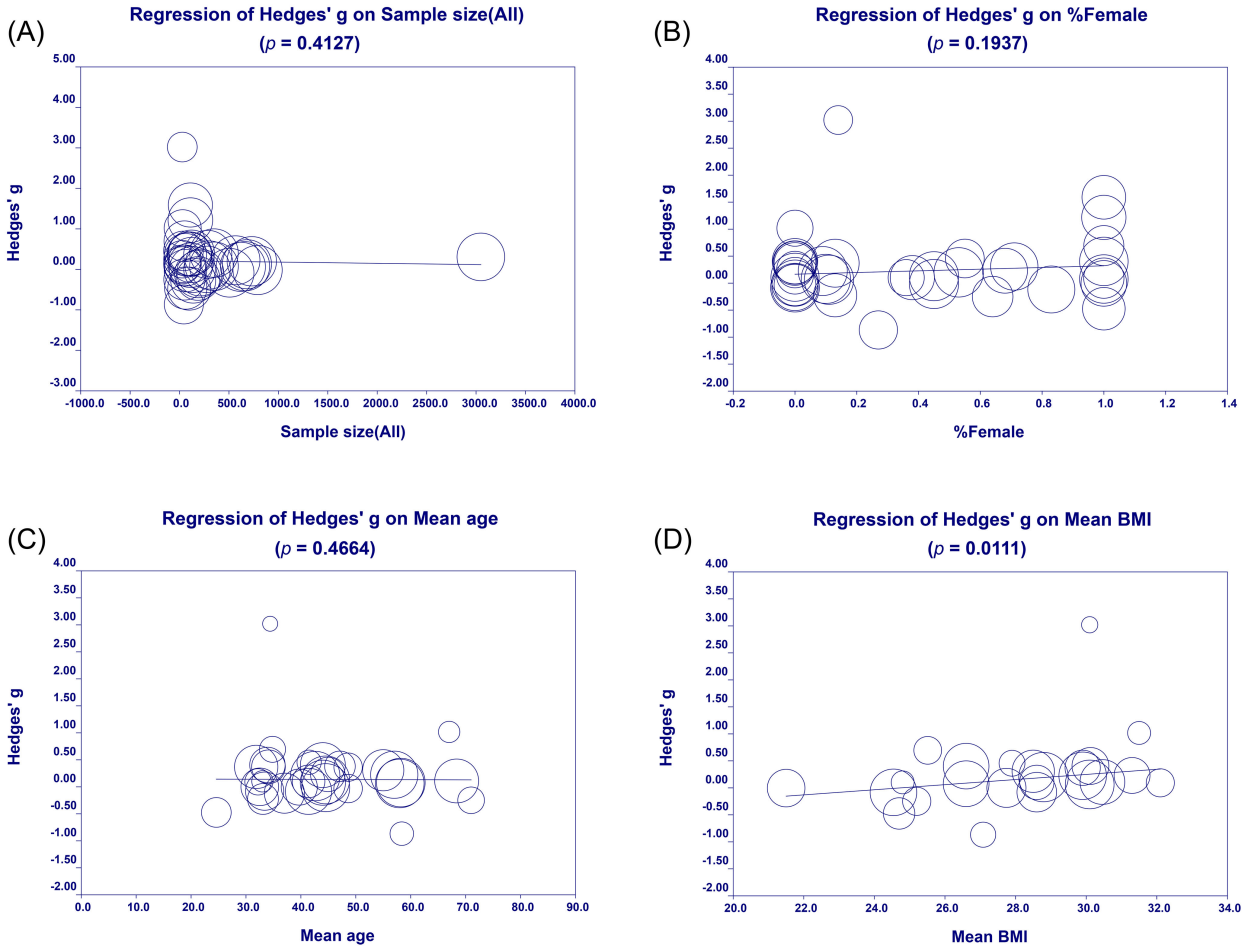
Meta-regression analysis of sample size **(A)**, %female **(B)**, mean age **(C)**, and mean BMI **(D)**.

Meta-regression analysis revealed that peripheral blood CRP levels were moderated by mean BMI (*p* = 0.0111). While sample size, % female, and mean age had no moderating effects on the outcome of the meta-analysis (**Figure 6**).

### 3.8 Sensitivity analysis

**Figure 7:**
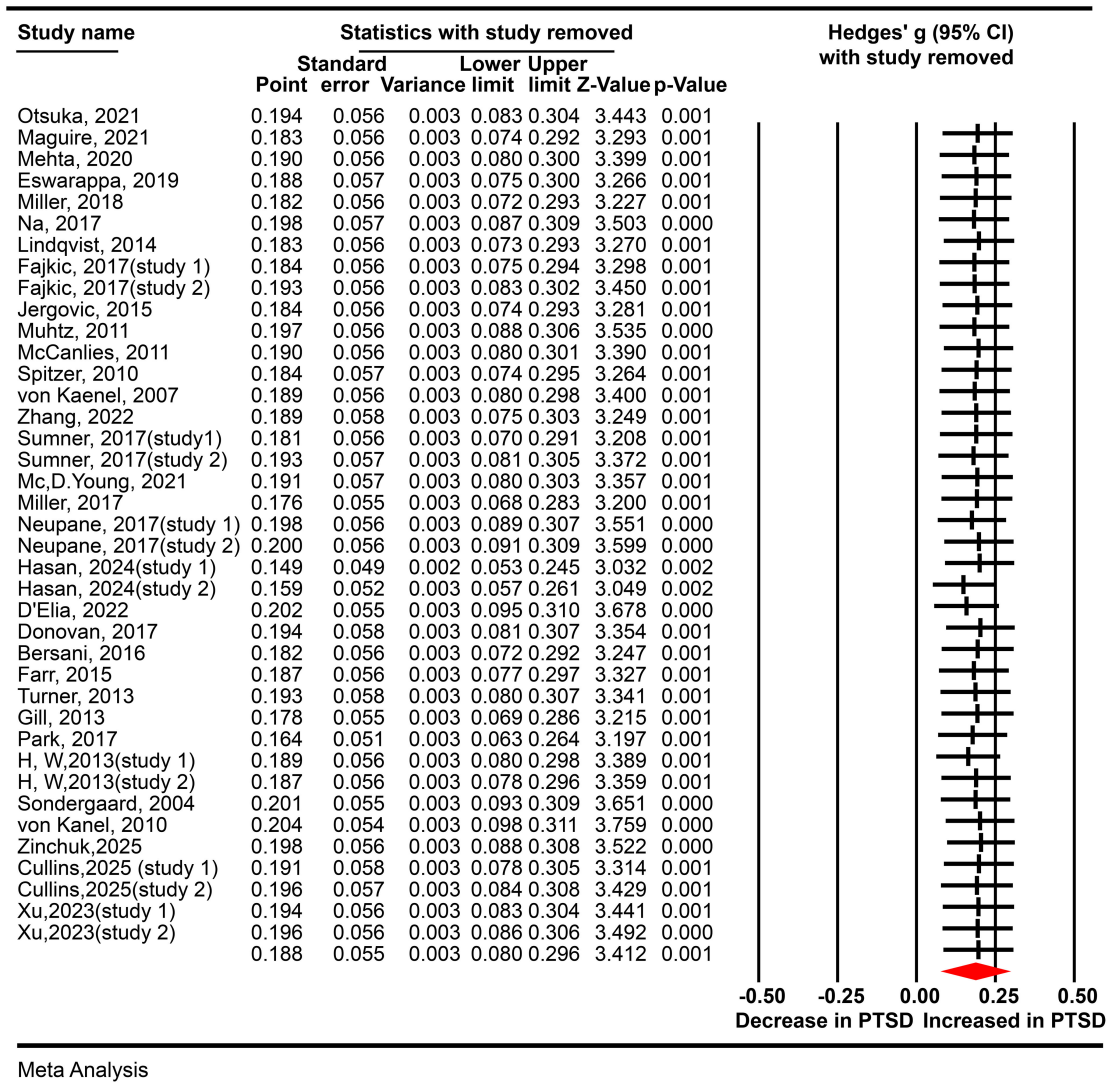
Sensitivity analysis of peripheral blood CRP level in PTSD patients versus controls.

To verify the effect of “one study removed” on the cumulative results, a sensitivity analysis was conducted. It was found that removing any individual study did not alter the pooled prevalence, confirming the robustness of our results (**Figure 7**).

### 3.9 Publication bias

**Figure 8:**
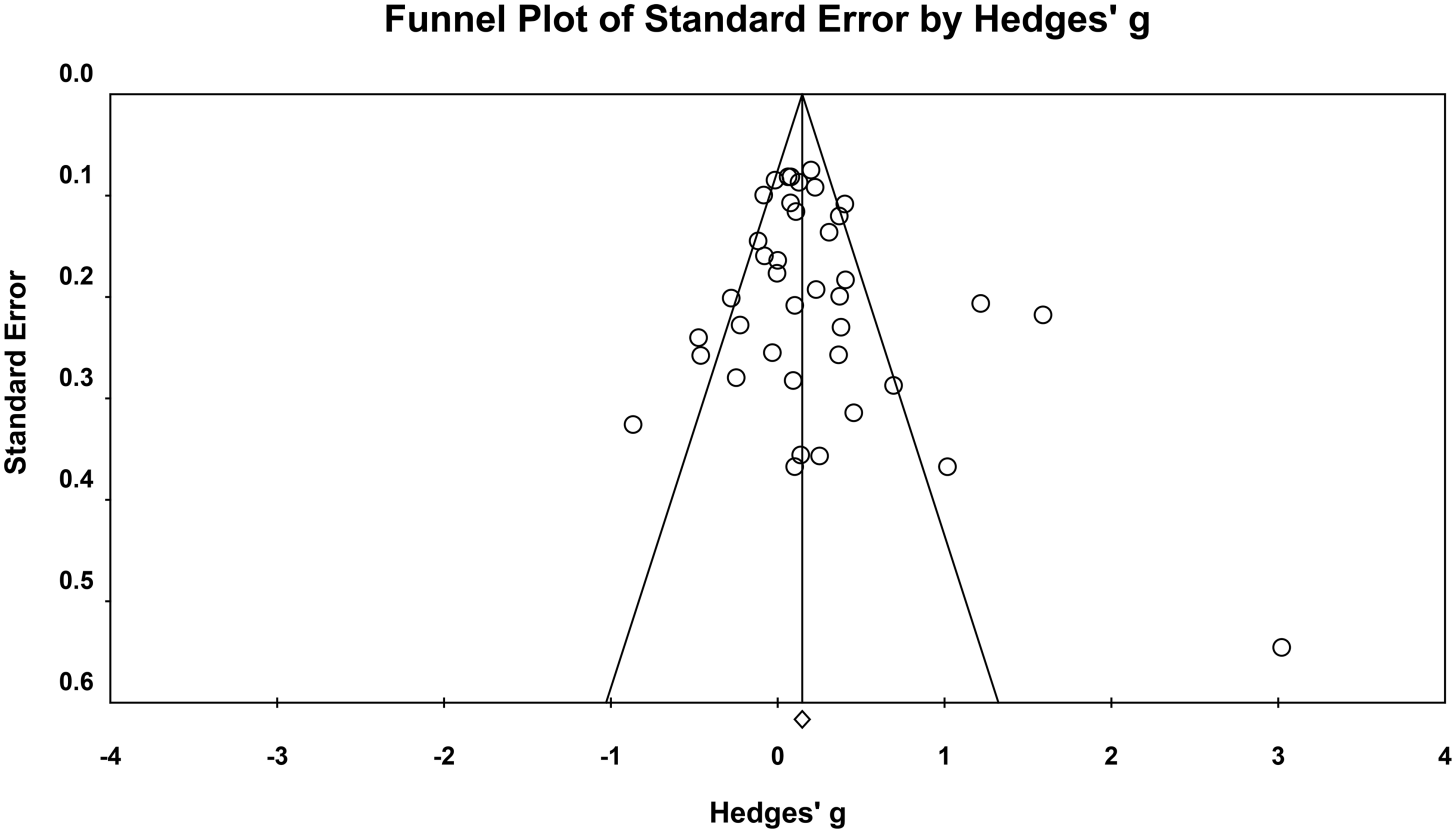
Funnel plot of publication bias.

The funnel plot of the analysis of peripheral blood CRP levels, as shown in **Figure 8**, revealed that there was no obvious publication bias existed, which was verified by Egger’s test (*p* = 0.22948).

## 4 Discussion

According to the meta-analysis, we observed a significant elevation in peripheral blood CRP levels in individuals with PTSD compared to controls. Importantly, sensitivity analysis confirmed the robustness of this finding even after excluding any individual study. As the presence of excessive heterogeneity among primary analysis, about 78.382%, may raise questions about the accuracy of our conclusions, we employed subgroup analysis, Galbraith plot and Baujat plot to identify the possible sources of heterogeneity. The results revealed that subgroup analyses were not effective in reducing heterogeneity in other groups, which exhibited moderate to high levels of heterogeneity, except for the male subgroup. This suggested that the heterogeneity of our analyses was influenced by multiple variables. The Galbraith plot identified 10 reports as statistical outliers contributing to the high heterogeneity, while the Baujat plot further specified that Hasan, 2024 (study 1), Hasan, 2024 (study 2), and Park, 2017 were the greatest contributors to this heterogeneity (41, 47). A systematic comparison of these 10 outlier reports with the remaining 29 reports revealed no consistent differences in terms of control type, gender, blood fraction, detection method, trauma type, NOS score, or potential confounding factors (including smoking, alcohol use, antidepressant use, comorbid depression, and comorbid somatic diseases). These studies were identified as outliers may primarily due to their extreme effect size estimates, which may be attributed to specific sample characteristics or unmeasured confounders within those individual studies, rather than a universal systematic bias. Importantly, the direction and statistical significance of the pooled effect remained unchanged after removing these outliers, indicating that the conclusion of elevated CRP in PTSD is robust. However, the magnitude of the effect size should be interpreted with caution, as the original pooled estimate (Hedge’g = 0.188) may represent a slight overestimation due to the influence of outlier studies. Future studies using standardized protocols will help further clarify the true effect size.

Subsequently, regression analysis was utilized to identify potential moderating factors. Meta-regression analysis revealed that sample size, % female, and mean age did not have any moderating effects in CRP concentrations among PTSD patients, while the mean BMI may serve as a confounding variable. It is not surprising that previous studies had already confirmed human adipose tissue expressed and released pro-inflammatory cytokines, thereby inducing low-grade inflammation, which lead to an increase in levels of CRP in overweight and obese adults (55, 56). The onset of PTSD is associated with immune dysfunction. Furthermore, the emergence of PTSD symptoms increased the risk of overweight and obesity and altered BMI trajectory over time (57). The existence of publication bias may result in incomplete research findings, exaggeration of study conclusions, and neglect of negative results (58). Our study demonstrated no significant evidence of publication bias, further confirming the reliability of our findings on the correlation between elevated peripheral blood CRP and PTSD.

In our research, a significant difference in CRP concentration was observed between PTSD patients and controls in serum samples, but not in plasma samples. We propose several reasons for this disparity. Firstly, We noticed that the number of clinical studies included in the serum group was twice as many as those in the plasma group, which helped mitigate the risk of type I and type II errors (59). Furthermore, Fiedorczuk et al. found that while the specificity of serum and plasma samples for CRP determination were similar, there are differences in sensitivity; specifically, the detection sensitivity for serum CRP was 35.85%, compared to 26.42% in plasma CRP (60). Additionally, CRP remains relatively stable in serum and can be preserved for up to 34 months (61). Consequently, the lack of statistical significance in the plasma subgroup does not necessarily indicate the absence of CRP elevation in PTSD, but rather suggests that the current evidence is insufficient to draw a definitive conclusion for plasma samples. Further large-scale primary studies with larger sample sizes, standardized protocols, and consistent choice of blood fraction are needed to validate these exploratory observations.

Among the studies included in this meta-analysis, the PTSD group exhibited significantly higher rates of smoking (32, 33), antidepressant use (32, 38, 44), and comorbid depression (32, 38, 42) compared to the control group. Given that previous studies have conclusively established that smoking (62, 63) and depression (64, 65) are independently associated with elevated CRP levels, and that antidepressants have been shown to modulate inflammatory markers (66), these factors constitute non-negligible confounding threats. Regrettably, limited by the data missingness and inconsistent measurement standards in the primary studies, we were unable to conduct further subgroup analyses or meta-regression analyses on the extracted confounding data to quantify their effects. Although our primary analysis indicated a significant association between PTSD and elevated CRP, we cannot rule out the possibility that the observed effect is at least partially attributable to these unadjusted confounders that are highly enriched in the PTSD population. This further underscores the urgent need for future primary studies to routinely and systematically report and ideally control for these critical variables.

Although the mechanistic link between peripheral inflammation and brain function in PTSD remains incompletely understood, emerging evidence suggests potential crosstalk between the peripheral and central immune systems. Under normal physiological conditions, peripheral CRP cannot cross the intact BBB (9). However, preclinical animal models indicate that severe stress can compromise BBB integrity by altering tight junctions (67, 68), damaging endothelial cells (69) and activating astrocytes and microglia (70), thereby potentially leading to neurological dysfunction. Alternatively, vagal afferent fibers can directly transmit peripheral inflammatory signals to the brain independent of alterations in BBB permeability (71, 72). Within the CNS, these transmitted signals could theoretically trigger neuroinflammation and microglial activation, leading to subsequent neuronal dysfunction that may contribute to PTSD symptomatology (73, 74). Multiple clinical studies have demonstrated elevated blood levels of inflammatory markers (e.g., IL-6, TNF-α, CRP and interferon (IFN)-γ) in PTSD patients (75–77), which is consistent with our current meta-analysis showing a significant elevation in peripheral CRP among PTSD patients.

During the literature screening process of this meta-analysis, we identified very few longitudinal studies, the included studies were predominantly cross-sectional, and none reported the critical variable of “time since trauma to blood collection”. From a physiological perspective, CRP, as an acute-phase reactant, can significantly elevate within hours to weeks following trauma (8, 78). Consequently, the CRP elevation observed in this study may reflect a physiological inflammatory response during the acute phase of trauma, persistent low-grade inflammation in the chronic PTSD state, or a mixed effect of both. This implies that we cannot ascertain whether the elevated CRP is a specific feature of PTSD as a psychiatric disorder, or a non-specific biological consequence of the traumatic event itself. Previous studies have indicated that CRP levels typically return to normal within weeks following acute trauma (79), whereas patients with chronic PTSD may exhibit elevated inflammatory markers lasting for years (13, 32, 38). However, due to the lack of individual-level data on time post-trauma, we were unable to verify this temporal pattern in the current meta-analysis. Therefore, it is imperative for future research to employ longitudinal designs, systematically record the time interval from trauma to blood collection, and dynamically track the trajectories of CRP level changes, thereby accurately elucidating the temporal relationship of inflammation in the onset and development of PTSD.

While the pooled effect size (Hedges’ g = 0.188) is statistically significant, it falls within the range of a small effect size according to conventional benchmarks (80). It is crucial to address the clinical relevance of this magnitude. Given the small effect size, CRP alone lacks the necessary sensitivity and specificity to serve as a standalone diagnostic or prognostic biomarker for PTSD, as the distributions of CRP levels between patients and controls overlap substantially (81) Moreover, CRP is a non-specific acute-phase reactant; its elevation is driven by numerous systemic confounders and reflects a generalized low-grade inflammatory state rather than a PTSD-specific pathological signature. Consequently, CRP alone is insufficient as a clinical biomarker. To enhance diagnostic accuracy and prognostic value, future research should focus on developing multi-marker panels that combine CRP with other more specific immune markers (e.g., IL-6, TNF-α) and neuroendocrine indicators, thereby moving beyond single, non-specific analytes toward comprehensive, multidimensional predictive models in PTSD (82).

To our knowledge, this is the largest systematic review and meta-analysis, focusing on evaluating peripheral blood CRP levels in PTSD patients compared to controls. The present meta-analysis encompasses 39 studies, more than double the number included in the previously largest meta-analysis, which consisted of 15 studies on CRP (83). While prior studies have established an increased odd of immune activation among PTSD patients, these studies primarily focused on alterations in multiple inflammation-related factors within PTSD patients (83, 84). The current meta-analysis systematically and thoroughly explored the association between peripheral blood CRP and PTSD, along with multiple plausible influencing factors. Our finding adds values by indicating that peripheral blood inflammatory biomarker CRP level is significantly higher in PTSD patients, further suggesting that peripheral blood CRP is a crucial part of the pathophysiology process of PTSD. Moreover, we identified that this correlation may be influenced by diverse factors such as blood fraction and mean BMI.

Although there are some important discoveries revealed by this study, there are also limitations. First, in our study, heterogeneity always existed, potentially attribute to uncontrolled variables (such as race, smoking, alcohol, and drug use) that have complex associations with immune alteration. Second, the correlation analysis between peripheral blood CRP and PTSD in this study provides us a comprehensive summary of results derived from cross-sectional studies. However, further longitudinal research is still needed to answer whether changes in CRP can be used to predict PTSD. Third, although we have included as many eligible literatures as possible, the language limitations have constrained us to only incorporate articles published in English, leading to the omission of certain crucial information. Fourth, the clinical significance of peripheral blood CRP levels, the causal relationship between elevated CRP levels and PTSD, and its potential as a diagnostic criterion for PTSD should be dialectically considered. Further basic researches are warranted to elucidate these inquiries.

## 5 Conclusion

The results of the present meta-analysis showed that elevated peripheral CRP level in PTSD patients, indicating inflammatory involvement in this population. Nevertheless, due to the cross-sectional design of the included studies and lack of data regarding the time intervals between trauma exposure and blood collection, the current findings cannot distinguish whether the observed CRP elevation is attributable to the trauma itself (acute inflammatory response) or specifically to PTSD as a psychiatric disorder (chronic inflammation). Future studies employing longitudinal designs to investigate CRP dynamics over time are crucial to distinguish the pathophysiological role of inflammation from the acute phase response.

## Data Availability

The original contributions presented in the study are included in the article/**Supplementary Material**. Further inquiries can be directed to the corresponding authors.
