# Supplementary material for: Peripheral blood levels of C-reactive protein in posttraumatic stress disorder: a systematic review and meta-analysis
Source: Front Psychiatry. 2026 Jul 30;17:1839701. doi: 10.3389/fpsyt.2026.1839701 (PMC13471148; doi:10.3389/fpsyt.2026.1839701)
Supplement: Supplementary file 1 [file DataSheet1.pdf]

Supplementary Table 1 Minor characteristics of included studies.

Reference	Sample size, n		Smoking, n (%)		Alcohol use, n (%)		Antidepressant, n (%)		Comorbid depression, n (%)		Comorbid somatic diseases, n (%)	
	Controls	PTSD	Controls	PTSD	Controls	PTSD		Controls	PTSD	Controls	PTSD	Controls
Otsuka, 2021, Japan	73	57	5(6.8%)	8(14.0%)	0 (0.0%)	9 (15.8%)	0 (0.0%)	30 (52.6%)	33 (57.9%)	0 (0.0%)	—	—
Maguire, 2021, UK	20	20	13(65%)	6(30%)	15(75%)	5(25%)	—	—	4(20%)	13(65%)	4(20.0%)[d]<br>0(0%)[e]	4(20.0%)[d]<br>0 (0%)[e]
Mehta, 2020, US	38	18	—	—	—	—	—	—	17.6±8.2[ak]	6.7±5.5 [ak]	—	—
Eswarappa, 2019, US	396	170	87(21.9%)	53(31.0%)	2.89±2.77 [al]	2.31±2.72 [al]	—	—	—	—	—	—
Miller, 2018, US	123	163	—	—	—	—	—	—	—	—	—	—
Na, 2017, Korea	368	139	—	—	—	—	—	—	—	—	—	—
Lindqvist, 2014, US	51	51	3(5.9%)	11(21.6%)	—	—	1(2.0%)	15(29.4%)	0(0.0%)	27(52.9%)	—	—
Jergovic, 2015, Croatia	32	69	5(15.6%)	37(53.6%)	14(43.8%)	14(20.3%)	—	47(68%)	5.3±5.8 [ak]	31.1±9.0 [ak]	—	—
Fajkić, 2017, Bosnia and Herzegovina (study 1)	30	30	0 (0.0%)	0 (0.0%)	0 (0.0%)	0 (0.0%)	0 (0.0%)	0 (0.0%)	0 (0.0%)	0 (0.0%)	0 (0.0%)	0 (0.0%)

(continued on next page)

Supplementary Table 1 *(continued)*

Reference	Sample size, n		Smoking, n (%)		Alcohol use, n (%)		Antidepressant, n (%)		Comorbid depression, n (%)		Comorbid somatic diseases, n (%)	
	Controls	PTSD	Controls	PTSD	Controls	PTSD	Controls	PTSD	Controls	PTSD	Controls	PTSD
Fajkić, 2017, Bosnia and Herzegovina (study 2)	30	30	0 (0.0%)	0 (0.0%)	0 (0.0%)	0 (0.0%)	0 (0.0%)	0 (0.0%)	0 (0.0%)	0 (0.0%)	0 (0.0%)	0 (0.0%)
Muhtz, 2011, Germany	25	25	0(0.0%)	1(4.4%)	17(68.0%)	16(64.0%)	—	—	0 (0.0%)	9(36%)	0 (0.0%)	0 (0.0%)
McCanlies, 2011, US	79	32	37(46.8%)	15(46.9%)	3.2±4.1[am]	2.8±3.2[am]	—	—	—	—	—	—
Spitzer, 2010, Germany	2994	55	1734(57.9%)	40(72.7%)	34.1±33.4[an]	24.6±32.4[an]	—	—	518(17.3%)	44(80%)	0.83±1.19[ao]	2.27±1.94[ao]
Zhang, 2022, US	415	316	134 (32.3%)	96 (30.4%)	—	—	—	—	—	—	—	—
von Känel,2007, Switzerland	14	14	8(57.1%)	7(50.0%)	6(42.9%)	7(50.0%)	—	—	—	—	—	—
Sumner, 2017, US (study 1)	175	174	10(5.7%)	16(9.2%)	54(30.9%)	82(47.1%)	10(5.7%)	62(35.6%)	15(8.7%)	43(25.4%)	—	—
Sumner, 2017, US (study 2)	175	174	7(4.0%)	16(9.2%)	60(34.5%)	82(47.1%)	15(8.6%)	62(35.6%)	5(2.9%)	43(25.4%)	—	—

(continued on next page)

Supplementary Table 1 *(continued)*

**Reference**	**Sample size, n**		**Smoking, n (%)**		**Alcohol use, n (%)**		**Antidepressant, n (%)**		**Comorbid depression, n (%)**		**Comorbid somatic diseases, n (%)**	
	**Controls**	**PTSD**	**Controls**	**PTSD**	**Controls**	**PTSD**	**Controls**	**PTSD**	**Controls**	**PTSD**	**Controls**	**PTSD**
Mc,D.Young, 2021, Australia	140	159	17.58±1.85[bp]	28.64±2.7[bp]	6.42±0.40[bl]	8.24±0.52[bl]	—	—	2.27±0.23[bq]	6.34±0.40[bq]	—	—
Miller, 2017, US	16	16	10(62.5%)	13(81.3%)	—	—	—	—	9.5±5.7[ar]	1.4±2.1[ar]	—	—
D'Elia, 2022, UK	41	58	—	—	—	—	—	—	0(0.0%)	56（96.5%）	—	—
Neupane, 2017, Nepal (study1)	47	32	—	21（65.6%）	—	25(86.2%)	—	—	—	22(68.8%)	—	—
Neupane, 2017, Nepal (study2)	107	32	—	21（65.6%）	—	25(86.2%)	—	—	—	22(68.8%)	—	—
Hasan, 2024, Iraq (study1)	55	55	0(0.0%)	0(0.0%)	0(0.0%)	0(0.0%)	0(0.0%)	0(0.0%)	0(0.0%)	0(0.0%)	0(0.0%)	0(0.0%)
Hasan, 2024, Iraq (study 2)	55	55	0(0.0%)	0(0.0%)	0(0.0%)	0(0.0%)	0(0.0%)	0(0.0%)	0(0.0%)	0(0.0%)	0(0.0%)	0(0.0%)
Donovan,2017, US	363	257	79(21.9%)	63(25.1%)	159(45.0%)	89(36.8%)	—	—	7(1.9%)	48(20.2%)	76(21.1%)	101(40.2%)

(continued on next page)

Supplementary Table 1 *(continued)*

**Reference**	**Sample size, n**		**Smoking, n (%)**		**Alcohol use, n (%)**		**Antidepressant, n (%)**		**Comorbid depression, n (%)**		**Comorbid somatic diseases, n (%)**	
	**Controls**	**PTSD**	**Controls**	**PTSD**	**Controls**	**PTSD**	**Controls**	**PTSD**	**Controls**	**PTSD**	**Controls**	**PTSD**
Bersani, 2016, US	65	56	4(6.2%)	9(16.1%)	—	—	2(3.1%)	17(30.4%)	13.36 ± 3.34[ak]	6.28 ± 4.03 [ak]	6(9.2%)[d] 1(1.5%)[e] 1(1.5%)[f] 0(0.0%)[g] 1(1.5%)[h] 4(6.2%)[i]	9(16.1%)[d] 3(5.4%)[e] 3(5.4%)[f] 1(1.8%)[g] 0(0.0%)[h] 2(3.6%)[i]
Farr, 2015, US	81	40	15 (19.2%)	23 (57.5%)	58 (75.3%)	25 (65.8%)	—	—	5 (6.5%)[k]	14(38.9%)[k]	—	—
Turner, 2013, US	433	230	—	—	3.4±3.0 [al]	3.1±3.1 [al]	—	—	64 (14.9%)	133 (58.3%)	46 (10.6%)[j] 59 (14.0%)[e]	40 (17.4%)[j] 44 (19.8)%[e]
Gill, 2013, US	24	26	2(8.3%)	5(19.2%)	—	—	—	—	—	—	—	—
Park, 2017, US	14	14	—	—	—	—	—	—	2(14.3%)	2(14.3%)	0(0.0%)	0(0.0%)
H, W,2013, US (study1)	15	15	6 (40%)	3 (20%)	5 (33%)	4 (27%)	5 (33%)	6 (40%)	0 (0%)	3 (20%)	—	—
H, W,2013, US (study2)	15	15	3 (20%)	3 (20%)	4 (27%)	4 (27%)	2 (13%)	6 (40%)	0 (0%)	3 (20%)	—	—
Söndergaard, 2004 Sweden	25	38	—	—	—	—	—	—	—	—	—	—

(continued on next page)

Supplementary Table 1 *(continued)*

Reference	Sample size, n		Smoking, n (%)		Alcohol use, n (%)		Antidepressant, n (%)		Comorbid depression, n (%)		Comorbid somatic diseases, n (%)	
	**Controls**	**PTSD**	**Controls**	**PTSD**	**Controls**	**PTSD**	**Controls**	**PTSD**	**Controls**	**PTSD**	**Controls**	**PTSD**
von Känel, 2010, Switzerland	29	15	2(7%)	3(20%)	5.3±6.3[am]	4.6±5.0 [am]	3(10%)	2(13%)	0 (0–1.5)[cs]	9 (5–11) [cs]	—	—
Zinchuk,2025, Russsia	95	95	0(0.0%)	0(0.0%)	0(0.0%)	0(0.0%)	—	—	95(100%)	95(100%)	0(0.0%)	0(0.0%)
Cullins, 2025, US (study 1)	502	180	—	—	0(0.0%)	180(100%)	—	—	—	—	—	—
Cullins, 2025, US, (study 2)	610	180	—	—	610(100%)	180(100%)	—	—	—	—	—	—
Xu, 2023, US, (study 1)	235	44	—	—	—	—	0(0.0%)	0(0.0%)	0(0.0%)	0(0.0%)	0(0.0%)	0(0.0%)
Xu,2023, US, (study 2)	158	52	—	—	—	—	0(0.0%)	0(0.0%)	0(0.0%)	0(0.0%)	0(0.0%)	0(0.0%)

— = not report; [a] Data represented Mean ± SD; [b] Data represented Mean ± SEM; [c] Data represented medians(inter-quartile range); [d] comorbid with hypertension; [e] comorbid with diabetes; [f] comorbid with stable angina; [g] comorbid with heart arrhythmias; [h] comorbid with prostate cancer; [i] comorbid with asthma/allergies; [j] comorbid with cardiovascular disease; [k] reflected by BDI scores; [l] reflected by AUDIT scores; [m] reflected by drinks/week; [n] reflected by alcohol (g/d); [o] reflected by Functional Comorbidity Index; [p] reflected by packs/year; [q] reflected by DASS21 Depression score; [r] reflected by HAM-D Total Score; [s] reflected by HADS depressive symptoms; SD, Standard Deviation; SEM, Standard Error of the Mean;

BDI, Beck Depression Inventory; AUDIT: Alcohol Use Disorders Identification Test; DASS-21, Depression Anxiety Stress Scale 21; HAM-D: The 17-item Hamilton Rating Scale for Depression; HADS, Hospital Anxiety and Depression Scale.

None of the included studies reported the time since trauma to blood collection.

Supplementary Table 2 Main characteristics of the studies removed by Galbraith plot.

Reference	PTSD diagnosis	Blood fraction	Detection method	Type of controls	Type of trauma	Sample size		Gender, Female/male		Age, Mean ± SD, years		BMI, Mean ± SD, kg/m^2		NOS Score
						Controls	PTSD	Controls	PTSD	Controls	PTSD	Controls	PTSD	
Na, 2017, Korea	IES-R-K	Plasma	Nephelometry	TC	—	368	139	0/368	0/139	37.67 ± 8.32	41.39 ± 7.28	24.03 ± 2.17	24.54 ± 2.20	6
Sumner, 2017, US (study 1)	DSM-IV PTSD	Plasma	Nephelometry	NTC	—	175	174	175/0	174/0	42.9 ± 4.8	44.0 ± 4.6	25.1 ± 5.5	26.6 ± 6.2	7
Miller, 2017, US	MINI, CAPS-5	Serum	—	TC	War combat	16	16	0/16	0/16	67.8 ± 1.6	67.0 ± 2.8	29.7 ± 5.6	31.5 ± 5.8	6
D'Elia, 2022, UK	CAPS-5	Serum	ELISA	NTC	Sexual violence	41	58	41/0	58/0	28±7.1	24.6±6.8	24.7±3.7	24.7±5.1	5
Neupane, 2017, Nepal (study2)	CIDI	Serum	—	TC	—	107	32	—	4/28	—	33.1 ± 7.9	—	—	6
Hasan, 2024, Iraq (study1)	PCL-5	Serum	Nephelometry	NTC	—	55	55	55/0	55/0	—	—	—	—	5
Hasan, 2024, Iraq (study 2)	PCL-5	Serum	Nephelometry	TC	Genocide-related events	55	55	55/0	55/0	—	—	—	—	5

(continued on next page)

Supplementary Table 2 *(continued)*

Reference	PTSD diagnosis	Blood fraction	Detection method	Type of controls	Type of trauma	Sample size		Gender, Female/male		Age, Mean ± SD, years		BMI, Mean ± SD, kg/m^2		NOS Score
						Controls	PTSD	Controls	PTSD	Controls	PTSD	Controls	PTSD	
Park, 2017, US	CAPS-IV, PCL-M	—	Nephelometry	TC	War combat	14	14	1/13	2/12	32.2 ± 1.5	34.4 ± 1.7	28.4 ± 1.2	30.1 ± 1.6	5
Söndergaard, 2004 Sweden	CAPS	Serum	Nephelometry	TC	War combat	25	38	—	—	18-48[a]	18-48[a]	—	—	5
von Känel, 2010, Switzerland	CAPS	Plasma	Flow cytometric cytokine assay	TC	Myocardial infarction	29	15	5/24	4/11	58.68±6.9	58.38±7.4	28.48±4.3	27.08±2.9	5

[a] Data represented as range; — = not report; SD, standard deviation; PTSD, posttraumatic stress disorder; BMI, body mass index; NOS score, Newcastle-Ottawa Scale score; NTC, non-trauma-exposed controls; TC, trauma-exposed controls; MINI, Mini-International Neuropsychiatric Interview; CAPS, Clinician-Administered PTSD Scale; PCL, PTSD Checklist-Civilian Version; DSM, Diagnostic and Statistical Manual of Mental Disorders; IES, Impact of Events Scale; CIDI, Composite International Diagnostic Interview; ELISA, Enzyme-linked immunosorbent assay.

None of the included studies reported the time since trauma to blood collection.

Supplementary Table 3 Minor characteristics of the studies removed by Galbraith plot.

Reference	Sample size, n		Smoking, n (%)		Alcohol use, n (%)		Antidepressant, n (%)		Comorbid depression, n (%)		Comorbid somatic diseases, n (%)	
	Controls	PTSD	Controls	PTSD	Controls	PTSD	Controls	PTSD	Controls	PTSD	Controls	PTSD
Na, 2017, Korea	368	139	—	—	—	—	—	—	—	—	—	—
Sumner, 2017, US (study 1)	175	174	10(5.7%)	16(9.2%)	54(30.9%)	82(47.1%)	10(5.7%)	62(35.6%)	15(8.7%)	43(25.4%)	—	—
Miller, 2017, US	16	16	10(62.5%)	13(81.3%)	—	—	—	—	9.5±5.7 [ar]	1.4±2.1 [ar]	—	—
D'Elia, 2022, UK	41	58	—	—	—	—	—	—	0(0.0%)	56（96.5%）	—	—
Neupane, 2017, Nepal (study2)	107	32	—	21（65.6%）	—	25(86.2%)	—	—	—	22(68.8%)	—	—
Hasan, 2024, Iraq (study1)	55	55	0(0.0%)	0(0.0%)	0(0.0%)	0(0.0%)	0(0.0%)	0(0.0%)	0(0.0%)	0(0.0%)	0(0.0%)	0(0.0%)
Hasan, 2024, Iraq (study 2)	55	55	0(0.0%)	0(0.0%)	0(0.0%)	0(0.0%)	0(0.0%)	0(0.0%)	0(0.0%)	0(0.0%)	0(0.0%)	0(0.0%)
Park, 2017, US	14	14	—	—	—	—	—	—	2(14.3%)	2(14.3%)	0(0.0%)	0(0.0%)
Söndergaard, 2004 Sweden	25	38	—	—	—	—	—	—	—	—	—	—

(continued on next page)

Supplementary Table 3 *(continued)*

Reference	Sample size, n		Smoking, n (%)		Alcohol use, n (%)		Antidepressant, n (%)		Comorbid depression, n (%)		Comorbid somatic diseases, n (%)	
	**Controls**	**PTSD**	**Controls**	**PTSD**	**Controls**	**PTSD**	**Controls**	**PTSD**	**Controls**	**PTSD**	**Controls**	**PTSD**
von Känel, 2010, Switzerland	29	15	2(7%)	3(20%)	5.3±6.3[am]	4.6±5.0 [am]	3(10%)	2(13%)	0 (0–1.5)[cs]	9 (5–11) [cs]	—	—

— = not report; [a] Data represented Mean ± SD; [c] Data represented medians(inter-quartile range); [m] reflected by drinks/week; [r] reflected by HAM-D Total Score; [s] reflected by HADS depressive symptoms; SD, Standard Deviation; HADS, Hospital Anxiety and Depression Scale.

None of the included studies reported the time since trauma to blood collection.
